# The Role of Dynamic miRISC During Neuronal Development

**DOI:** 10.3389/fmolb.2020.00008

**Published:** 2020-01-31

**Authors:** Bharti Nawalpuri, Sreenath Ravindran, Ravi S. Muddashetty

**Affiliations:** ^1^Centre for Brain Development and Repair, Institute for Stem Cell Science and Regenerative Medicine (Instem), Bangalore, India; ^2^School of Chemical and Biotechnology, Shanmugha Arts, Science, and Technology and Research Academy (SASTRA) University, Thanjavur, India; ^3^Manipal Academy of Higher Education, Manipal, India

**Keywords:** miRNA, miRISC, neuronal development, translation regulation, neuronal activity

## Abstract

Activity-dependent protein synthesis plays an important role during neuronal development by fine-tuning the formation and function of neuronal circuits. Recent studies have shown that miRNAs are integral to this regulation because of their ability to control protein synthesis in a rapid, specific and potentially reversible manner. miRNA mediated regulation is a multistep process that involves inhibition of translation before degradation of targeted mRNA, which provides the possibility to store and reverse the inhibition at multiple stages. This flexibility is primarily thought to be derived from the composition of miRNA induced silencing complex (miRISC). AGO2 is likely the only obligatory component of miRISC, while multiple RBPs are shown to be associated with this core miRISC to form diverse miRISC complexes. The formation of these heterogeneous miRISC complexes is intricately regulated by various extracellular signals and cell-specific contexts. In this review, we discuss the composition of miRISC and its functions during neuronal development. Neurodevelopment is guided by both internal programs and external cues. Neuronal activity and external signals play an important role in the formation and refining of the neuronal network. miRISC composition and diversity have a critical role at distinct stages of neurodevelopment. Even though there is a good amount of literature available on the role of miRNAs mediated regulation of neuronal development, surprisingly the role of miRISC composition and its functional dynamics in neuronal development is not much discussed. In this article, we review the available literature on the heterogeneity of the neuronal miRISC composition and how this may influence translation regulation in the context of neuronal development.

## Introduction

MicroRNAs (miRNAs) are among the most studied and discussed regulators of gene expression in the last two decades. The reason they have attracted so much attention is because of their specificity in targeting and versatility in their function. These small RNAs (~22 nt long) recognize their targets through a “seed sequence” (forming 5–8 base pair match on target mRNA) and lead to a rapid decrease in the corresponding protein level by translation repression and/or degradation of mRNA (Baek et al., 2008; Guo et al., 2010; Bartel, 2018; Duchaine and Fabian, 2019). Due to their sequence specificity and requirement of short nucleotide stretch for base pairing, single miRNA can target several mRNAs (Hashimoto et al., 2013). miRNAs control gene expression at multiple levels and affect every aspect of cellular functions in multicellular organisms (Janga and Vallabhaneni, 2011). More recently, there has been an explosion of studies on miRNAs due to their potential implication in diagnostic and therapeutic approaches in multiple human diseases ranging from cancer to neurodegenerative disorders (Peng and Croce, 2016; Ramakrishna and Muddashetty, 2019). The effect of miRNAs on translation depends on the recruitment of a complex which includes several protein components. This protein complex together with miRNA forms the microRNA induced silencing complex (miRISC) which is essential to execute the miRNA function (Krol et al., 2010; Fabian and Sonenberg, 2012). While our appreciation of the role of miRNAs in biology is ever-increasing, there is a significant gap in our understanding of the function of individual components of the miRISC and its compositional heterogeneity.

Unlike small interfering RNA (siRNA), miRNA mediated translation repression is a multistep process. Several studies have indicated a clear correlation between decreased protein levels and the degradation of mRNAs targeted by miRNAs (Baek et al., 2008; Guo et al., 2010). However, a less appreciated fact is that the kinetics of this process is highly variable for different sets of mRNAs (Baek et al., 2008; Hendrickson et al., 2009; Guo et al., 2010). In all cases, translation repression precedes mRNA degradation and now it has been established that in many instances, translation repression can be reversed (Bhattacharyya et al., 2006; Muddashetty and Bassell, 2009; Djuranovic et al., 2011; Kute et al., 2019). The whole logic of a complex multistep process to induce translation repression could be to facilitate the reversibility of this process. This makes miRNA mediated regulation a powerful tool for the spatiotemporal regulation of gene expression. The kinetics and reversibility of miRISC depend to a large extent on its composition. Hence, it is very important to identify and classify these proteins into the core miRISC components and the additional/ancillary ones. The core miRISC facilitates the default miRISC pathway which includes, translation repression followed by deadenylation, decapping, and degradation of mRNA (Huntzinger and Izaurralde, 2011; Fabian and Sonenberg, 2012). Additional factors generally regulate the kinetics of this pathway which could also result in stalling of the process at any of the stages of the default pathway. More importantly, these factors could provide reversibility to the miRISC mediated translation repression and make it a more dynamic process to regulate gene expression.

Reversibility of miRNA mediated translation repression is likely to happen in all kinds of cells during the growth, division, differentiation or in response to stress and external cues (Bhattacharyya et al., 2006; Jafarifar et al., 2011; Bellon et al., 2017; Patranabis and Bhattacharyya, 2018). This mechanism is particularly apt for neurons where compartmentalized and cue dependent translation is evolved to a great extent. There are many reviews that discuss the role of miRNAs in neuronal development and plasticity (Siegel et al., 2011; Ye et al., 2016). But the dynamic property of miRISC greatly depends on its protein composition (Huntzinger and Izaurralde, 2011; Fabian and Sonenberg, 2012). In this review, we explore the protein composition of miRISC and how it determines the function of miRNAs in neurons. We particularly focus on different developmental stages of the nervous system where the alteration of this composition could fine-tune the gene expression to equip the response of neurons to external cues.

## The Dynamic Nature of miRISC Function and Its Potential for Reversibility

**Figure 1:**
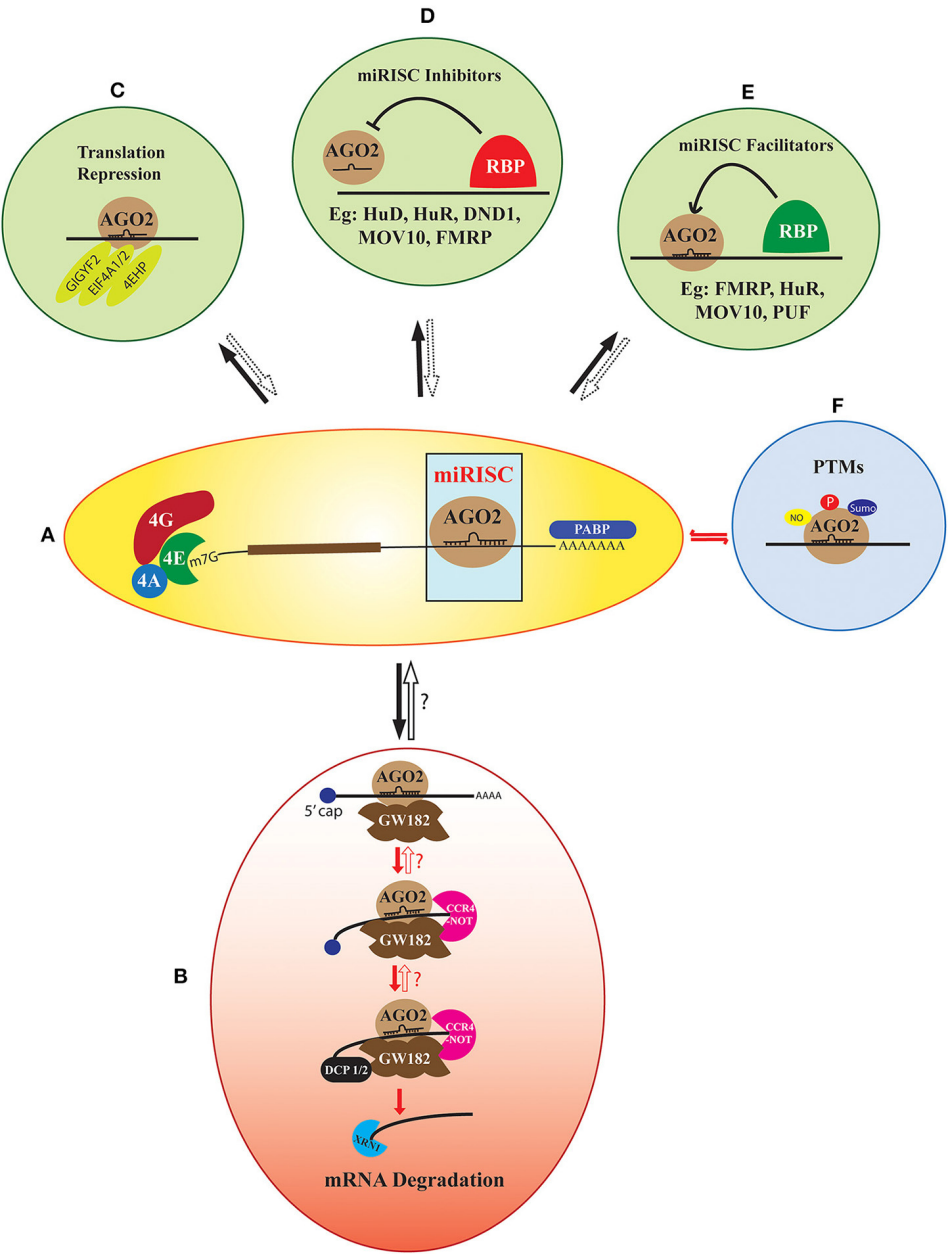
The heterogeneity in miRISC effector function. AGO2 recruited on a target mRNA along with its cognate miRNA form the core of miRISC **(A)**. The default function of this complex is deadenylation and decapping-dependent degradation of the target mRNA. This pathway involves the sequential assembly of proteins like GW182, CCR4-NOT, DCP1/2, and XRN1 to the core miRISC **(B)**. However, this multi-step assembly could be stalled and potentially reversed at several of these steps. Apart from the default pathway, the core miRISC can also associate with other translation regulators like GIGYF2, EIF4A1/2, and 4EHP, which lead to translation repression of the target mRNA without degradation **(C)**. A large number of RNA Binding Proteins (RBPs) like FMRP, MOV10, Hu proteins, Pumilio, and DND1 either facilitate or inhibit miRISC assembly on an mRNA **(D,E)**. Another added layer of heterogeneity is contributed by the post-translational modifications (PTMs) of the miRISC protein components. Modifications like phosphorylation, sumoylation, and nitrosylation are reported both on the core miRISC protein AGO2 or the accessory members **(F)**. The fate of the target mRNA is determined by all the above-mentioned factors concerning miRISC along with the specific cellular context. However, the precise mechanisms of the reversible miRISC function, as well as its inhibitors and facilitators are not completely known.

The miRISC function has two primary components, target recognition and silencing of the targeted transcript. The miRNA-element confers specificity to the target, while the gene expression repression functions are carried out by different protein components of miRISC (Fabian and Sonenberg, 2012; Duchaine and Fabian, 2019) (Figure 1). The primary protein partner of miRISC is the Argonaute protein. Argonaute proteins are an indispensable part of miRISC complex as they bring together miRNAs and mRNAs to form the core of miRISC (Höck and Meister, 2008; Bartel, 2018) (Figure 1A). The rest of the miRISC comprises of a diverse set of proteins, such as mRNA degradation machinery components, scaffolding proteins, and many different RNA binding proteins (RBPs), which assemble with the core miRISC to form compositionally diverse miRISCs. This composition, in turn, appears to be a crucial determinant of the kinetics and the outcome of the miRISC function (Dallaire et al., 2018).

As a default mechanism, the formation of miRISC on an mRNA leads to its degradation in a multistep pathway (Fabian et al., 2010; James et al., 2012; Jonas and Izaurralde, 2015) (Figure 1B). Integrated results from transcriptome-wide analysis coupled with ribosome profiling, and mass spectrometry techniques have revealed mRNA destabilization as the primary result of miRNA function in multiple cell lines (Baek et al., 2008; Hendrickson et al., 2009; Guo et al., 2010). This lead to the widely accepted (what we call “default”) model of miRISC function, which involves miRNA mediated inhibition of translation, followed by deadenylation, decapping, and degradation of target mRNA (Bazzini et al., 2012; Djuranovic et al., 2012). However, now there is sufficient evidence to suggest that this default pathway can be significantly altered by additional factors which can stall and reverse the miRNA mediated inhibition at multiple steps (Ashraf et al., 2006; Bhattacharyya et al., 2006; Kedde et al., 2007; Banerjee et al., 2009; Muddashetty et al., 2011; Kute et al., 2019). This reversible regulation provides flexibility to the system for rapid modulation of protein levels on different cues, bypassing the need for new mRNA transcription. In developmental contexts, such as oogenesis, early embryogenesis and in neurons, translational repression is preferred over irreversible mRNA decay. This helps to stably maintain a pool of specific mRNAs whose temporally controlled expression is critical in development or synaptic plasticity. Thus, we emphasize that the data from transcriptome-wide and ribosome-profiling studies should not be generalized, as this may ignore the importance of the structural and functional heterogeneity of miRISC in cell type and development specific contexts.

## Diverse miRISC Composition and Its Regulation Brings About the Dynamic Functions

Argonaute (AGO) associates with miRNAs and load them on to their cognate target mRNAs to form the core of the miRISC (Figure 1A). Now there is good amount of data is available on the structure and function of distinct domains of Argonaute protein (Song et al., 2004; Hall, 2005; Peters and Meister, 2007; Höck and Meister, 2008; Hutvagner and Simard, 2008; Boland et al., 2011; Cenik and Zamore, 2011; Meister, 2013; Swarts et al., 2014; Dayeh et al., 2018). The Argonaute family of proteins are evolutionarily conserved with multiple paralogues present in diverse species (Swarts et al., 2014). The mammalian genome encodes four Argonaute proteins, of which only AGO2 possesses the endonucleolytic activity. AGO2 is the most abundant and is also best studied among its paralogues with respect to miRNA function (Carmell et al., 2004; Tuschl et al., 2004). However, all four Argonaute proteins can efficiently perform miRNA mediated translation repression and act in an overlapping and redundant manner (Su et al., 2009; Wang et al., 2012). Among the four AGO proteins, AGO3 and AGO4 are sparsely studied mostly due to the low abundance of these proteins in adult tissues (Su et al., 2009; Wang et al., 2012; Völler et al., 2016).

In addition to miRNA mediated post-transcriptional gene silencing, AGO proteins are reported to perform various moonlighting functions in other subcellular compartments. Nuclear localized AGO proteins regulate transcriptional silencing, epigenetic modulation, alternative splicing, and DNA repair (Huang and Li, 2014; Ross and Kassir, 2014). The function of nuclear-localized AGO complexes has been previously reviewed and hence will not be discussed further in this review (Huang and Li, 2014; Ross and Kassir, 2014; Kalantari et al., 2016; Sharma et al., 2016; Liu et al., 2018).

The imperfect complementarity of metazoan miRNA-mRNA pairs prevents the endonucleolytic activity of AGO2, leading to the requirement of additional factors for the effective degradation of target mRNA. In its default mode, the scaffolding protein GW182 is first recruited on to the core miRISC, which in turn facilitates the binding of deadenylation and decapping enzymes as well as exonucleases to form large miRISC complex (Fabian and Sonenberg, 2012). This complex primarily results in the degradation of targeted mRNA (Figure 1B). GW182, a critical component of this pathway, is a large Glycine Tryptophan (GW) repeats containing protein. The structure and function of different domains of GW182 have been critically reviewed (Ding and Han, 2007; Baillat and Shiekhattar, 2009; Chekulaeva et al., 2009; Eulalio et al., 2009; Bazzini et al., 2012; Zielezinski and Karlowski, 2015; Niaz and Hussain, 2018). GW182 binds to AGO2 through its N-terminal domain following which it interacts with Polyadenylate Binding Protein (PABP) on the 3′ end of mRNA. This interaction would interrupt the circularization of mRNA leading to translation repression (Zekri et al., 2009, 2013; Moretti et al., 2012). The C-terminal silencing domain of GW182 further recruits deadenylase complexes Poly(A)-Nuclease (PAN) deadenylation complex (PAN2-PAN3) and Carbon Catabolite Repressor 4 (CCR4)-Negative on TATA (NOT) to PABP free mRNA leading to deadenylation (Behm-Ansmant et al., 2006; Chekulaeva et al., 2011; Fabian et al., 2011). In most animal systems, the deadenylated mRNA is decapped by Decapping protein 1/2 (DCP1/2)-followed by mRNA degradation through 5′-3′ Exoribonuclease 1 (XRN1) (Rehwinkel et al., 2005; Behm-Ansmant et al., 2006).

The temporal sequence and the relative contribution of translation repression and mRNA degradation in the canonical mode of miRISC function are not fully understood. However, majority of the studies suggest that translation repression is followed by mRNA degradation unless cellular degradation machinery is inhibited (Bazzini et al., 2012; Béthune et al., 2012; Djuranovic et al., 2012; Jonas and Izaurralde, 2015). The coupling between translation repression and mRNA degradation depends on relative kinetics of translation repression, deadenylation, and decapping process. Hence, we hypothesize that translation repression by miRISC can be effectively uncoupled from mRNA degradation thus providing an effective way for reversible translational regulation. Compared to the mRNA degradation function, the mechanisms contributing to translational repression by miRNAs are more heterogeneous and less understood. In the following sub-section, we describe several well-studied mechanisms of miRISC mediated translation repression.

Apart from the default mechanism, miRISC is shown to function via several alternate mechanisms (Figure 1C). These involve the interaction of core miRISC with components of the translation machinery and diverse RNA binding proteins. Translation machinery proteins shown to be central in such alternate pathways include Eukaryotic Initiation Factor 4F (eIF4F) complex subunit eIF4A (Meijer et al., 2013; Fukao et al., 2014; Fukaya et al., 2014), and the helicase DDX6 (Kamenska et al., 2016). The interaction of miRISC with these proteins either precludes the normal assembly of the translational machinery or recruits additional elements, such as eIF4E homolog 4EHP. miRISC is also shown to interact with the components of the ribosomal complex, such as Receptor for Activated C Kinase 1 (RACK1), Ribosomal Protein S14 (RPS14), Ribosomal Protein L5 (RPL5), and Ribosomal Protein L11 (RPL11) (Chan and Slack, 2009; Jannot et al., 2011; Liao et al., 2013).

The activity of miRISC is heavily modulated by its interactions with multiple RBPs (Fukao et al., 2015; Gardiner et al., 2015; Loffreda et al., 2015). These RBPs either associate directly with miRISC to influence its function, or they modulate miRISC activity by binding close to the miRNA binding site in the target mRNAs. Proteins like Fragile X Mental Retardation Protein (FMRP), MOV10 Moloney leukemia virus 10 (MOV10), Ataxin 2, and Fused in Sarcoma (FUS) directly associate with core miRISC to influence its downstream effector functions (Banerjee et al., 2009; McCann et al., 2011; Muddashetty et al., 2011; Sudhakaran et al., 2014; Zhang T. et al., 2018) (Figures 1D,E). The association of FMRP with miRISC was earlier contested as one of the report has shown that FMRP does not associate with miRISC and is an important component of stress granules (Didiot et al., 2008). However, further later reports showed the association of FMRP with core miRISC protein AGO2 (Jin et al., 2004; Muddashetty et al., 2011; Kute et al., 2019). RBP Pumilio positively modulates miRISC activity by unwinding mRNA 3′UTR to promote miRNA binding (Friend et al., 2012). Multiple members of TRIM-NHL family of proteins interact with AGO to positively modulate miRISC repressive activity (Hammell et al., 2009; Schwamborn et al., 2009) (Figure 1E). In contrast, proteins, such as Dead end protein homolog 1 (DND1), Heterogeneous Nuclear Ribonucleoprotein L (hnRNPL), APOBEC3G and Hu family proteins inhibit miRISC function either by competing with miRISC target site on mRNA or by preventing functional miRISC formation (Bhattacharyya et al., 2006; Kedde et al., 2007; Jafarifar et al., 2011; Liu et al., 2012) (Figure 1D). Notably, most of the miRISC repression modulated by different RBPs is shown to be reversible. Although many of these studies investigate the interaction of various RBPs with AGO2, the association of these RBPs with GW182 and how that may influence miRISC reversibility remains unclear.

**Figure 2:**
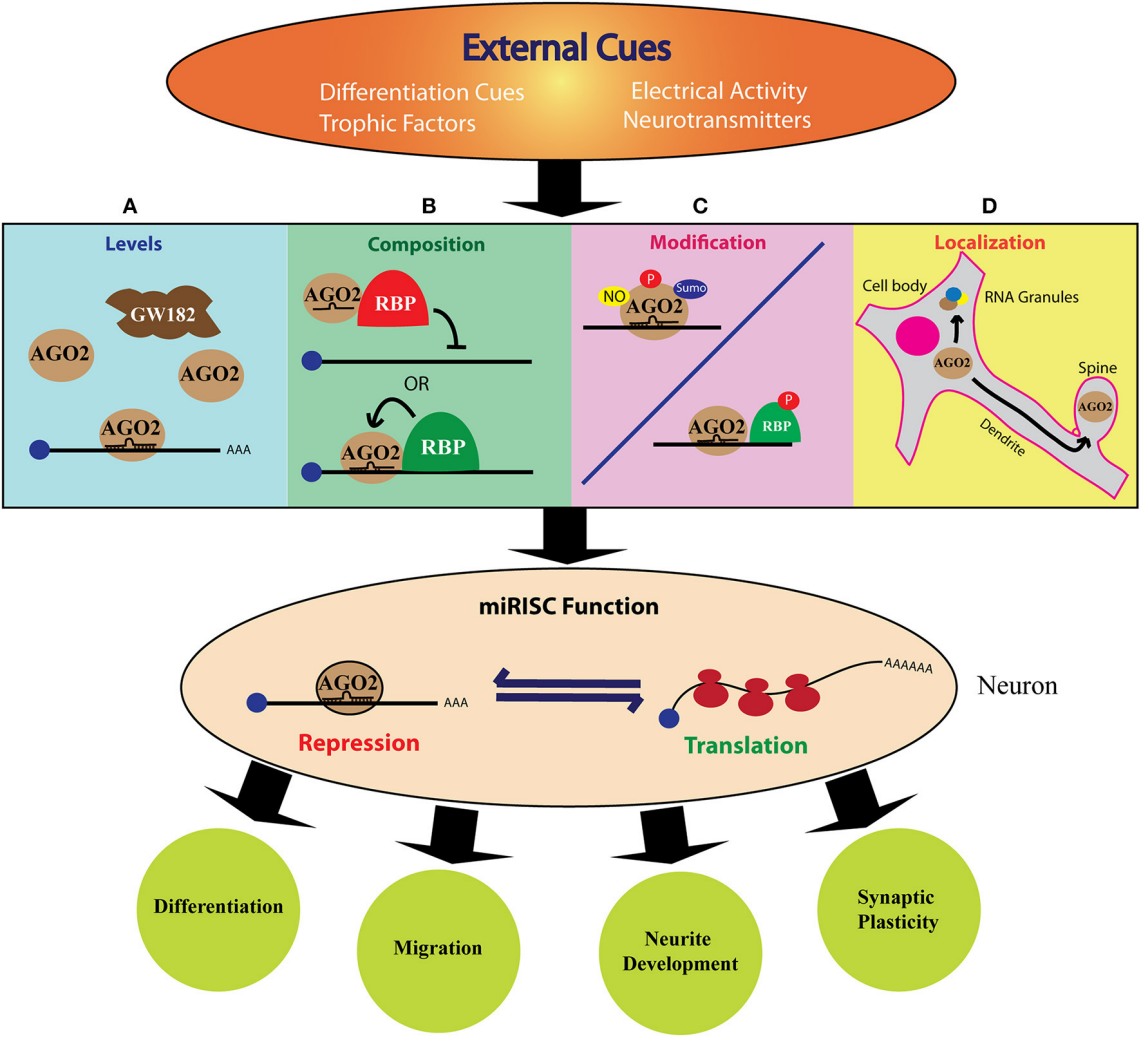
The role of miRISC in cue mediated protein synthesis during neurodevelopment. The above figure shows how different external cues can modulate miRISC function to fine-tune neurodevelopment. The specific, rapid, local, and reversible mode of miRNA action qualifies miRISC mediated mechanism as an excellent mode for extrinsic regulation of neuronal protein synthesis. External signals, such as trophic factors or neuronal activity can induce changes in the levels **(A)**, composition **(B)**, post-translational modification **(C)**, or localization **(D)** of one or more of miRISC components. This can alter the fine balance between translation and repression/decay of the target mRNA. This, in turn, leads to specific changes in the cellular proteome either globally or locally and enable the cell to differentiate, migrate, grow neurites or form synapses.

The work described until now clearly establishes the wide compositional diversity of miRISC. The important question is how this diversity influences the effector function of miRISC? The broad mechanisms by which the composition and external cues modulate the miRISC function is shown in Figure 2. These factors include cellular proliferation status, specific spatiotemporal context, and different extracellular signals. External signals modify the miRISC function through different mechanisms. One such crucial mechanism is post-translational modifications (PTMs) (Figures 1F, 2C). The core miRISC component AGO2 is modified by phosphorylation, sumoylation, prolyl hydroxylation, and ubiquitination at multiple sites (Figure 1F). Such PTMs regulate divergent aspects of AGO2 function, such as stability, miRNA binding, mRNA association, and interaction with GW182 (Qi et al., 2008; Rüdel et al., 2011; Horman et al., 2013; Martinez and Gregory, 2013; Sahin et al., 2014; Bridge et al., 2017; Chinen and Lei, 2017; Golden et al., 2017). Another mechanism of regulating miRISC is via protein-protein interactions. AGO2-GW182 interaction is highly regulated by various extracellular cues (Olejniczak et al., 2013, 2016; Wu et al., 2013; La Rocca et al., 2015; Bridge et al., 2017; Rajgor et al., 2018). AGO2 interaction with RBPs, as well as the modulation of miRISC activity by RBPs are shown to be regulated by different signals like hypoxia, arsenite stress, mTOR signaling, and neuronal activity (Bhattacharyya et al., 2006; Banerjee et al., 2009; Jafarifar et al., 2011; Muddashetty et al., 2011; Sosanya et al., 2013). Cell-specific regulation of miRISC composition was recently demonstrated by work from Simard's lab (Dallaire et al., 2018). The structure of the miRNA binding site on mRNA also regulates the miRISC (Zhang K. et al., 2018). Additionally, specific subcellular localization of miRNA and miRISC machinery can also confer structural and functional dynamicity to miRISC (Trabucchi, 2019). These mechanisms generally have an overlapping regulatory function, as in, a post-translational modification can affect the level or localization of a particular miRISC protein.

## Cue Mediated Protein Synthesis in Neurons: Potential Role of miRISC Dynamics and Function

**Figure 3:**
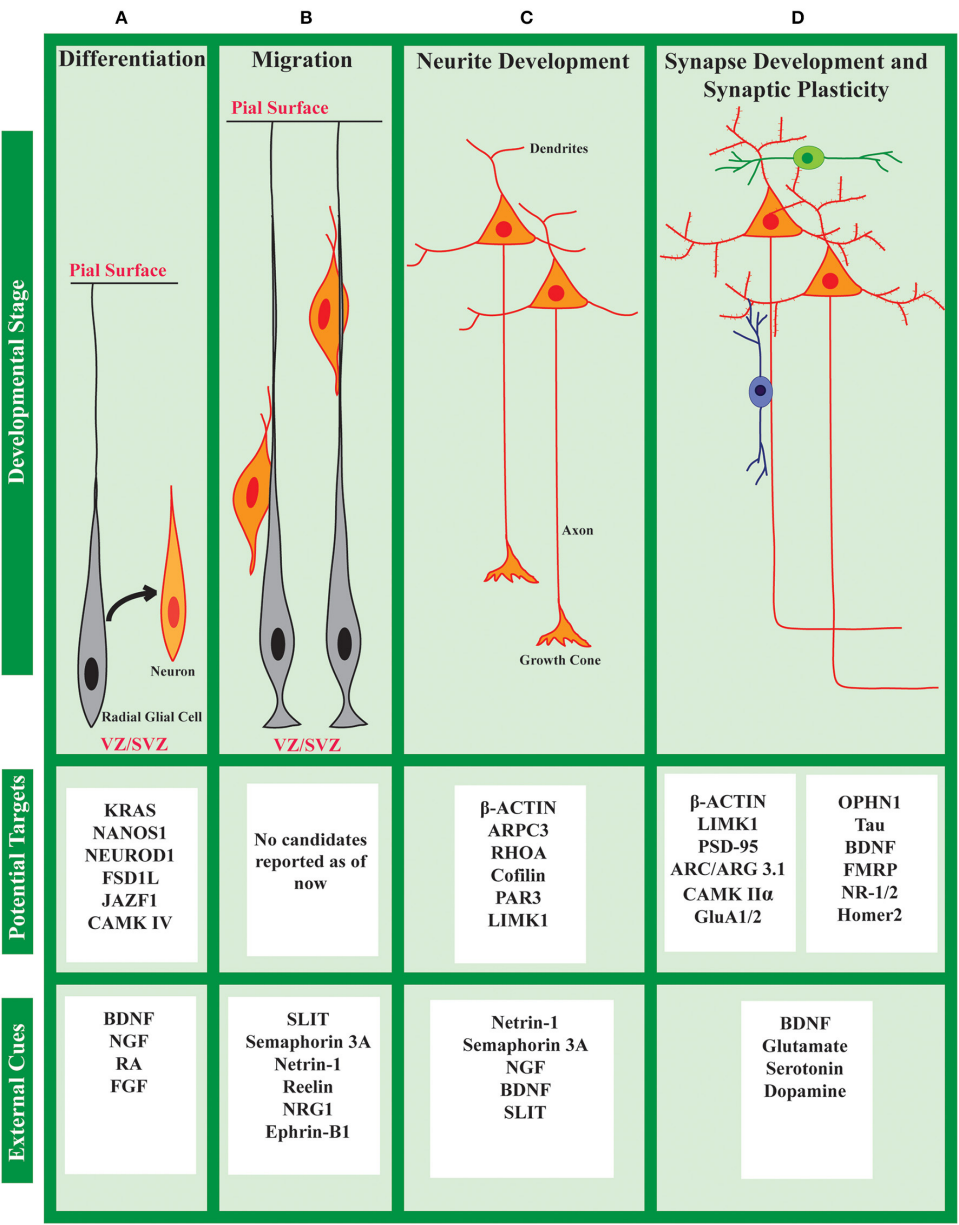
Cue induced protein synthesis in neuronal development: cues and targets. The development of a neuron can be divided into different stages **(A–D)** as shown above. At each stage, the neuron responds to a set of external cues that regulates its development. Along with activating transcription, these cues also affect the translational status of specific mRNAs to fine-tune the global/local proteome. Several such candidates are listed above, although specific links between cues and candidates are not known in many cases.

Spatiotemporal regulation of the intracellular proteome is important for every cell in multicellular organisms. But, it is of critical significance to neurons due to their highly polarized organization, and the specificity of the connections. There is substantial de-centralization of protein synthesis in neurons which is well-established through the demonstration of the presence of the translational machinery, as well as, a wide repertoire of mRNAs, in various neuronal compartments (Zelená, 1970; Steward and Levy, 1982; Torre and Steward, 1992; Chicurel et al., 1993; Rao and Steward, 1993). Apart from that, neurons also have evolved mechanisms to tightly regulate protein synthesis in response to neuronal activity. There is now evidence to show the significant contribution of activity-mediated protein synthesis in neurodevelopment and synaptic plasticity (Kang and Schuman, 1996; Kauderer and Kandel, 2000; Campbell and Holt, 2001; Wu et al., 2005; Yao et al., 2006; Costa-Mattioli et al., 2009; Blair et al., 2017; Ravindran et al., 2019). Defects in this regulation are also thought to be the underlying pathophysiology of several neurodevelopmental disorders. There are several excellent recent reviews on these concepts (Steward and Schuman, 2003; Swanger and Bassell, 2013; Kim and Jung, 2015). Since this review is focused on the role of translational regulation during neurodevelopment, we have listed out different external cues affecting the stages of neuronal development as well as the different proteins reported to be translationally regulated during specific stages in Figure 3. However, it is to be noted that a signal to the target-protein link is not established for many of the cases.

The specific, rapid, local, and reversible mode of miRNA action qualifies miRISC mediated mechanism as an excellent choice for regulating neuronal protein synthesis. Moreover, the reversibility of miRISC is a well-suited mechanism for neuronal functions because, in contrast to targeting the mRNA for degradation, neurons often require to store mRNA in a repressed state either for local transport or for cue mediated translation. As depicted in Figure 2, the dynamic nature of miRISC in neurons could be achieved in multiple ways. First, the expression of different miRISC components can be regulated during neuronal development and synaptic activity (Figure 2A). This can dictate the type of miRISC formed in a particular scenario. Additionally, the expression of the miRISC proteins can be regulated locally at specific neuronal compartments, such as dendrite, spine or axonal growth cone. Extracellular factors, such as axon guidance cues, neurotrophic factors, and neurotransmitter signals can also influence miRISC composition (Figure 2B) by altering the post-translational modification (Figure 2C) and sub-cellular localization (Figure 2D) of miRISC components. Not surprisingly, much of the literature investigating the role of RBPs in miRISC functions have utilized neuronal model systems.

## Dynamic miRISC in The Regulation of Neuronal Development

Development of the nervous system in humans is a complex and prolonged phenomenon starting as early as 3 weeks of gestation and continuing until adolescence (Stiles and Jernigan, 2010; Elshazzly and Caban, 2019). It involves an orchestrated network of cell fate changes as well as cellular and tissue morphogenesis driven by cell-intrinsic and activity-dependent mechanisms (Katz et al., 1992). Development of the nervous system begins with neurulation—the process by which a segment of ectodermal cells specialize to form the neural plate which invaginates inside and fuses to form the neural tube. The neural tube is a hollow tube running in the anteroposterior axis of the embryo which gives rise to the central nervous system. The rostral end of the tube forms the brain and the caudal region forms the spinal cord. The peripheral nervous system is derived from the neural crest cells, which are formed near the dorsal line of fusion of the neural tube. Rodents also follow the same pattern of neurodevelopment. Although different kinds of cells are involved in the formation of the neuronal network, in this review, we have limited our area of interest to the development of the neurons. The development of a typical neuron in the central nervous system (CNS) can be broadly classified into the following overlapping phases: neurogenesis, subtype specification, neuronal migration, neurite growth, synaptogenesis, and synaptic pruning. In rodents, the first three phases are predominantly pre-natal and later phases continue into the post-natal period. In the next section, we will be discussing different miRISC complexes formed and their role in neuronal development.

### Neurogenesis and Differentiation

In rodents, neurogenesis starts at around E10 and is complete by about E18 (Semple et al., 2013). A part of the neuronal population is derived directly from radial glial cells (RGC), which in turn is derived from neuroepithelial cells (NEC) (Figure 3A). Indirect neurogenesis occurs from RGCs through the production of intermediate progenitors (IP). The site of neurogenesis is close to the ventricular zone (VZ). The process of sub-type specification mostly coincides with neurogenesis. Early in the development, neural progenitor cells can give rise to a wide variety of neuronal populations. Later on, they become more sub-type specific (Taverna et al., 2014; Jiang and Nardelli, 2016).

The prevailing view in the field describes transcriptional regulation as the predominant gene regulatory program during neuronal differentiation. However, emerging studies have established the importance of post-transcriptional mechanisms in neuronal differentiation (Kraushar et al., 2014, 2015; Blair et al., 2017; Sugiyama et al., 2017; Hwang et al., 2018; Zahr et al., 2018; Mohammad et al., 2019; Tahmasebi et al., 2019). Multiple miRNAs have been shown to regulate distinct aspects of neuronal differentiation (Stappert et al., 2015). Studies from over a decade have established the pivotal role of miRISC proteins and their composition in regulating neuronal differentiation. To begin, the core miRISC protein AGO has been shown to undergo paralogue switching from AGO1 to AGO2 during nervous system development (Juvvuna et al., 2012). The study demonstrated a more potent regulation of miR-124 induced neuronal differentiation via AGO1-miRISC as compared to AGO2-miRISC. Additionally, the authors associated the increased fractional abundance of AGO2 with the progressive shortening of miRNA 3′ end during nervous system development. Thus, the authors suggested an interesting possibility where 3′ end trimming of miRNA regulates their target specificity across neuronal development. AGO1 is also reported to associate with TRIM-NHL protein Tripartite Motif-containing protein 32 (TRIM 32) to prevent neuronal differentiation in mouse cortical progenitor neurons. TRIM-32 associates with AGO1 to increase the activity of specific miRNAs, such as Let-7a. In C. elegans, Trim-NHL protein NHL-2 is shown to function as a cofactor for miRISC in regulating neuronal cell fate changes (Hammell et al., 2009). TRIM-32 was shown to associate with miRISC protein DDX6 for regulating neuronal differentiation (Nicklas et al., 2015). The mechanistic details of TRIM-NHL proteins interactions with AGO and its effect on the miRNA pathway are yet to be elucidated. However, findings across species suggest that they have a clear role in modulating the miRISC pathway during neuronal differentiation. Another recent study demonstrated that Nerve Growth Factor (NGF) induced differentiation of PC12 cells requires phosphorylation of AGO2 at Tyr-529 (Patranabis and Bhattacharyya, 2016). This results in the release of Let-7a miRNA from AGO2 and subsequent activation of Let-7a target KRAS leading to neuronal differentiation. Ectopic expression of P-body proteins, AGO2 or GW182 was sufficient to induce differentiation pointing to a direct link between miRISC activity and cell fate decisions (Patranabis and Bhattacharyya, 2018). An important role of miRISC was recently described in the regulation of the quiescent state in adult neuronal stem cells (Katz et al., 2016). This study demonstrated a non-canonical nuclear role of AGO and GW182 associated miR-9 complex in regulating neuronal differentiation. Alteration of the nuclear/cytoplasmic ratio of this miR-9-RISC resulted in deregulation of NSC quiescent state. An important future direction is to delineate whether such a mechanism also regulates neuronal differentiation during early development.

Multiple other miRISC interacting proteins, such as FMRP, Pumilio, and Hu family of proteins have been shown to play an indispensable role in regulation of neuronal differentiation (Kasashima et al., 1999; Anderson et al., 2000; Akamatsu et al., 2002; Castren et al., 2005; Tervonen et al., 2009). However, since all these proteins have been shown to regulate translation independent of miRISC, their relative contribution in the context of miRISC is yet to be elucidated. Thus, the current evidence indicates that even though transcriptional changes may be the predominant factor driving neurogenesis/differentiation, translational control through miRISC modulation also plays a critical role in driving these processes.

### Neuronal Migration

From the site of differentiation close to the VZ, neurons migrate to their final destination near the periphery of the neural tube by a process called radial migration. Initially in development, this occurs through somal translocation. Later, radial glial cells guide the neuronal migration (Figure 3B). As a general rule, neurons follow an “inside-out” migratory pattern, as the first formed neurons occupy the deeper layers and the later formed neurons migrate past them to form the superficial layers. In the developing neocortex, this migratory pattern leads to the formation of a six-layer architecture. Several external cues like Reelin, Ephrin, Semaphorin 3A, Netrin-1, etc. are known to regulate this process of migration (Jiang and Nardelli, 2016; Buchsbaum and Cappello, 2019). The importance of translation regulation in neuronal migration is sparsely studied. However, considering the directional migration of a polarized cell, it is very likely that local translation could be of importance. Indeed, some miRISC interactors are shown to play an important role in migration. For example, FMRP is shown to regulate neuronal migration and cortical positioning by affecting the multipolar to bipolar transition in migrating neurons (La Fata et al., 2014). Using mass spectrometry-based study, Weinmann et al. (2009) identified Importin-8 (IPO-8) as an AGO interacting protein. It interacts with AGO1-4 and is required for the recruitment of AGO complex onto a large set of target mRNAs. IPO-8 is also required for the radial migration of cortical projection neurons (Nganou et al., 2018). However, there is no direct evidence for the role of cue-induced miRISC modulation in neuronal migration. Technical limitations may be an important hurdle to study this. Developments in the field of organoid culture might bridge this gap in the future.

### Polarization and Neurite Growth

Neurons are heavily polarized cells with characteristic sub-type specific dendritic and axonal arbor. In most multipolar neurons in the cerebral cortex, several neurites outgrow from the soma and one of them is soon differentiated to be the axon. The rest of the neurites grow as dendrites. The axons generally grow faster and develop large growth cones at their tips which guide them to the putative targets (Figure 3C). Both axonal and dendritic growth is heavily dependent on various guidance cues and trophic factors (Dickson, 2002; Arikkath, 2012; Jiang and Nardelli, 2016).

Studies from the last three decades have established the essential role of cue mediated local translation in axonal growth and axonal regeneration. Subsequently, studies have also explored the role of miRISC composition in the regulation of the above-mentioned mechanism. Multiple components of miRISC machinery are shown to localize in developing axons and growth cones (Hengst et al., 2006; Murashov et al., 2007; Dajas-Bailador et al., 2012). A recent study has shown the co-localization of core miRISC (AGO2 and miRNAs) with mitochondria at axonal branch points and growth cone of peripheral nerve axons (Gershoni-Emek et al., 2018). miRISC protein FMRP has been shown to regulate Semaphorin 3A mediated growth cone collapse (Li, 2009). Another study has shown that FMRP associates with miR-181d and AGO2 to mediate axonal delivery of *Map1b* and *Calm1* mRNAs (Wang et al., 2015). The authors further demonstrated that NGF mediated the local release of *Map1b* and *Calm1* mRNA from FMRP containing repressive granules, resulting in axonal translation. This study provides an important proof of principle showing the importance of cue regulated miRISC composition in axonal development. It is important to determine whether miRISC also associates with other axonal transport proteins for regulating the transport of translationally repressed mRNA targets.

The importance of miRISC mediated translation regulation is not investigated extensively in mature axons. This is primarily due to the apparent absence of protein synthesis in mature axons (Kim and Jung, 2015; Biever et al., 2019). However, recent studies have demonstrated axonal translation and its importance in synaptic plasticity (Shigeoka et al., 2016; Younts et al., 2016; Scarnati et al., 2018), thereby providing a platform for future studies of miRISC involvement in translation regulation in mature axons.

miRISC is also shown to play an important role in regulating axonal regeneration upon injury. Expression of several miRISC components as AGO2, FMRP, GW182, and DCP1 is shown to be induced in axonal varicosities upon neuronal injury (Wu et al., 2011). This increased expression was accompanied by an increased co-localization of GW182 and DCP1 in axons. An interesting insight from these studies is that lesion-induced up-regulation of miRISC proteins was seen only in axons, leaving the possibility that the injury causes an axon-specific translation of miRISC proteins.

In contrast to its important role in axonal development, the importance of translation regulation in dendritic development is yet to be established. However, recent studies showing the essential requirement of ribosomes and different RBPs in dendrite morphogenesis, have hinted toward an important role of translation regulation in dendritic development (Vessey et al., 2010; Olesnicky et al., 2014; Antonacci et al., 2015; Slomnicki et al., 2016; Ravindran et al., 2019). Due to the apparent lack of studies on translation regulation in dendrite development, the function of miRISC and its compositional dynamics is very sparsely studied. Till date, only one study has shown the importance of cue dependent modulation of miRISC composition for the regulation of dendritic growth (Huang et al., 2012). The authors showed that BDNF increases the interaction of miRISC scaffolding protein GW182 with AGO2 and DDX6. This increased interaction regulates BDNF induced translation and dendritic growth (Huang et al., 2012). The authors also demonstrated that perturbation of GW182 function abolished BDNF induced dendritic growth. Interestingly, the authors did not see any effect of GW182 function on basal dendritic growth. However, this study was performed on DIV14 cultured neurons whereas most of the dendritic growth happens between DIV 3–12 in neuronal culture. Hence, it is important to determine whether a similar regulatory mechanism functions during the early stages of dendritic development.

Though the role of translation regulation in dendritic development is underexplored, several miRISC proteins are known to regulate dendrite development. In drosophila, dead box helicase Me31B (DDX6 homolog) interacts with FMRP and AGO to regulate dendritic elaboration in larval sensory neurons (Barbee et al., 2006). The study also showed that the effect of Me31B on dendritic growth is correlated with its ability to mediate translation repression via miRNA. Various miRISC components, such as DCP1A, AGO2, DDX6, and GW182 was shown to localize to dendrites to form dendritic P bodies like structures (dlP) (Barbee et al., 2006; Cougot et al., 2008). miRISC modulators, such as FMRP, MOV10, and Pumilio was also shown to regulate dendrite morphogenesis. However, this does not necessarily implicate their involvement with miRISC for the regulation of dendritic development, and this forms an exciting area for future studies.

Another correlative evidence suggesting miRISC as a regulator of dendrite morphogenesis is that various miRISC proteins show peak expression or activity during an intensive period of dendritogenesis. The decapping activator DCP1a shows development associated changes in its phosphorylation status (Blumenthal et al., 2009). The fractional abundance of the non-phosphorylated form of DCP1a increases in the adult brain. DCP1 phosphorylation is known to affect its activity as a decapping activator by regulating DCP1a-DCP2 interaction (Chiang et al., 2013). Thus, an interesting possibility is that the reduction in phosphorylated DCP1a could result in a reduced decapping and degradation efficiency of miRISC. Different subunits of the CCR4-NOT deadenylase complex also demonstrate a specific neurodevelopmental profile (Chen et al., 2011). The expression of subunits CNOT1, CNOT2, CNOT3, CNOT6, CNOT8, and CNOT9 drastically reduces during brain development. The functional implication of reduced expression of CCR4-NOT complex subunits at this developmental stage has not been determined. However, one can hypothesize that reduced CNOT complex expression along with reduced DCP1 phosphorylation might lead to a reduction in the proportion of degradatory miRISC in the adult brain. In summary, while the role of extrinsic modulation of miRISC is well-established during axonal development, its role in dendrite morphogenesis needs further explorations.

### Synapse Formation, Maintenance, and Synaptic Pruning

As the axonal and dendritic processes meet their respective partners, further they mature to form pre- and post-synaptic compartments, respectively (Figure 3D). Neuronal activity and mutual chemical signaling between the two partners are critical for the establishment of a synapse. Typically, multiple weak connections are established initially between two partner neurons. Eventually, the network is refined by strengthening some connections and eliminating the rest (Yogev and Shen, 2014; Südhof, 2018). This process of synaptic pruning is a persistent feature of post-natal development until adulthood.

Studies have demonstrated the presence of entire protein synthesis machinery along with ribosomes at developing and mature synapses (Steward et al., 1988). Several studies have established the important function of protein synthesis in synapse formation, maintenance and pruning (Burry, 1985; Steward, 1987; Steward et al., 1988; Scheetz et al., 2000; Munno and Syed, 2003; McCann et al., 2007). Given the important role of cue regulated protein synthesis, it is very likely that miRISC mediated regulation has a critical role in synaptogenesis. The core miRISC component AGO2 has been shown to co-localize with miRNA processing enzyme dicer in the postsynaptic compartment (Lugli et al., 2005; Hanus and Schuman, 2013). FMRP-AGO miRISC was shown to play an important role in the regulation of synaptic growth at larval NMJ (Neuromuscular junction) in drosophila. Drosophila harboring mutations in dFMR1 or AGO1 (Drosophila homolog of mammalian AGO2) shows synaptic overgrowth phenotype along with an increased number of synaptic boutons (Jin et al., 2004). A recent study shows evidence for the important role of miRISC protein, decapping activator HPat1 in regulating synaptic growth at the glutamatergic NMJ in Drosophila larvae (Pradhan et al., 2012). The study demonstrated the presence of strong synaptic hyperplasia at the NMJ in HPat mutants. Further, the study showed that HPat genetically interacts with the catalytic subunit of the deadenylase complex (twin/CCR4) and the miRNA pathway (AGO1) to control bouton formation. These studies hint at the need to further explore the role of cue mediated miRISC modulation during synaptogenesis and pruning.

### Synaptic Plasticity

Synaptic plasticity is the mechanism by which specific patterns of synaptic activity results in changes in synaptic strength (Figure 3D). Different types of synaptic plasticity exist in the central nervous system which play an important role in the regulation of memory formation and consolidation. Multiple studies have established the unequivocal role of protein synthesis in different forms of synaptic plasticity (Kang and Schuman, 1996; Pfeiffer and Huber, 2006; Sutton and Schuman, 2006; McCann et al., 2007; Cajigas et al., 2010; Younts et al., 2016). Cue mediated modulation of miRISC composition and its importance in driving local protein synthesis is also widely studied in the context of synaptic plasticity (Ashraf et al., 2006; Banerjee et al., 2009; Muddashetty et al., 2011; Rajgor et al., 2018; Kute et al., 2019).

miRISC components have been shown to form heterogeneous and dynamic granules in neurons (Cougot et al., 2008; Zeitelhofer et al., 2008; Oh et al., 2013; Luchelli et al., 2015). These neuronal miRISC granules have been shown to re-localize to distal dendritic sites and post-synapse on synaptic activity (Cougot et al., 2008; Oh et al., 2013). Furthermore, synaptic stimulation has been shown to result in rapid loss of miRISC protein AGO2 from these neuronal miRISC granules (Cougot et al., 2008). A recent study demonstrated the important role of XRN1 containing synaptic granules in translation regulation downstream of different glutamate receptors (Luchelli et al., 2015). The authors characterized the presence of novel XRN1 granules at synapse named as synaptic XRN1 (SX) bodies, which did not contain any canonical P body components like DCP1a, EDC4, or RCK/P54. Further, it was shown that the synaptic concentration of SX bodies is affected by synaptic stimulation and is inversely correlated with the rate of local translation. Interestingly, disruption of SX bodies by XRN1 knockdown resulted in abrogation of NMDAR mediated translation repression response.

Compositionally distinct miRISC complexes have been shown to form in response to synaptic stimulation. These complexes play a crucial role in regulating the translation of specific mRNA downstream of synaptic activity. Drosophila miRISC protein AGO1, dFMR1, and Me31B were shown to interact with Ataxin-2 to regulate long term olfactory habituation and translation of CaMKII mRNA (McCann et al., 2011; Sudhakaran et al., 2014). Synaptic activation has been shown to modulate miRISC composition by affecting expression, post-translational modifications, subcellular localization and interactions of different miRISC constituents.

Work from Gary Bassell's lab established FMRP phosphorylation as a reversible switch in regulation miRISC mediated repression of specific target mRNAs (Muddashetty et al., 2011). This study demonstrated that phosphorylated FMRP associates with AGO2 and miR-125a to inhibit PSD-95 translation. mGluR activation causes FMRP phosphorylation, leading to dissociation of inhibitory miRISC complex from FMRP-bound PSD95 and its subsequent translation. Further studies suggested that mGluR induced dephosphorylation of FMRP facilitates its ubiquitination and proteasomal degradation (Nalavadi et al., 2012). Important questions like the interaction of FMRP with miRISC protein GW182 and how FMRP prevents miRISC mediated target degradation require further investigation. Surprisingly, the role of FMRP-miRISC mediated translation regulation downstream of NMDAR signaling remains relatively unexplored. Albeit, a recent study from our lab have suggested an important role of AGO2-MOV10-FMRP contain miRISC complexes in regulating NMDAR mediated translation response (Kute et al., 2019). This work shows that dissociation of MOV10 from AGO2 complexes is required for NMDAR mediated translation activation of a specific subset of mRNAs, such as PTEN and PSD95. NMDAR induced dissociation of MOV10 complexes from AGO2 requires phosphorylated FMRP. Consequentially, NMDAR induced translation of these specific target mRNAs is defective in FMR1 KO animals.

Modulating the phosphorylation of AGO2 is shown to be a mechanism employed for regulating NMDAR mediated translation response and spine dynamics (Antoniou et al., 2014; Rajgor et al., 2017, 2018). Hanley's lab has identified AGO2 as an interacting partner of PDZ and BAR domain-containing scaffolding protein: Protein Interacting with C Kinase 1 (PICK1) in neurons (Antoniou et al., 2014). PICK interaction with AGO2 has shown to localize AGO2 to recycling endosomes, and suppress AGO2 mediated miRNA repression (Antoniou et al., 2014). The authors showed that NMDAR induced long term depression (LTD) mediated increase in Ca^2+^ results in a reduction of PICK-AGO2 interaction, leading to AGO2 dissociation from endosomes. This led to a subsequent increase in AGO2 mediated repression by dendritically localized miRNAs like miR-134 and miR-138 (Rajgor et al., 2017). A later study from the same lab demonstrated that NMDAR stimulation results in increased interaction of AGO2 with GW182 and DDX6. The increased interaction was mediated by S387 phosphorylation of AGO2 via the AKT pathway and was essential for NMDAR induced spine shrinkage. On the contrary, another study has reported that NMDAR activation leads to the dephosphorylation of AGO2 at S387 (Paradis-Isler and Boehm, 2018). This dephosphorylation was shown to induce degradation of AGO2 resulting in de-repression of miRNA mediated silencing and subsequent spine growth and maturation. The explanation for such contradictory results could be the temporally distinct effect of NMDAR stimulation on synaptic translation and hence miRISC composition, an area needs to further explored. The above reports suggest that a difference in stimulation paradigm used and the time of experimentation after NMDAR stimulation (immediate vs. post-recovery experimentation), determines the phosphorylation response of AGO2 and resulting in distinct spine phenotypes.

Different miRISC components are degraded downstream of neuronal activity to mediate synaptic plasticity. For example, long term memory formation in drosophila NMJ is regulated by activity mediated rapid degradation of miRISC protein Armitage (Ashraf et al., 2006). Activity mediated Armitage degradation is required for the translation of synaptic mRNA CaMKII, which is further required for the induction of long-term potentiation (LTP). A similar mechanism has been described in hippocampal neurons where NMDAR mediated proteasomal degradation of miRISC protein MOV10 (Armitage homolog) leads to the translation of several synaptic mRNAs, such as LIMK1, CaMKIIα, and Lyp1a (Banerjee et al., 2009). These were among the first reports to show the importance of miRISC composition in reversible regulation of synaptic translation. The expression of deadenylase CCR4-NOT transcription complex subunit 7 (CNOT7) is regulated downstream of synaptic activity (Rajgor et al., 2017). The expression of CNOT7 drastically decreases upon glycine induced long-term potentiation of cultured hippocampal neurons (Rajgor et al., 2017). Furthermore, shRNA mediated knockdown of CNOT7 is shown to disrupt LTP, suggesting an important role of modulation of CNOT7 expression in LTP. Currently, it has not been established whether this reduction in CN0T7 also results in reduced activity of canonical miRISC. In short, miRISC reveals to be an important hub of regulation downstream of different synaptic signaling pathways and is crucial in mediating synaptic plasticity.

## Conclusions

The work we discussed in this review highlight the importance of dynamic miRISC in normal neuronal development. Several miRISC proteins are shown to have a significant role during distinct stages of neuronal development as summarized in Table 1. But what is lacking is the understanding to link the role of these proteins in the context of miRISC function to their physiological impact. To get this insight, it is important to consider miRISC as a dynamic translation modulator rather than just as mRNA degradation machinery. In this review, we have analyzed how individual RBPs association with core miRISC may alter the kinetics and translation and degradation of a specific subset of mRNAs. Making a distinction between the “core” and “ancillary” components of miRISC helps us understand the role of many RBPs refereed to be part of miRISC as individual components or in combination. The key intention of this review is to reanalyze the role of the microRNA associated RNA binding proteins involved in neuronal development by their potential contribution to the dynamic nature of miRISC function.

**Table 1:** The role of miRISC proteins in neurodevelopment.

**Differentiation**	**Migration**	**Neurite development**	**Synapse development and synaptic plasticity**
**AGO2** (Patranabis and Bhattacharyya, 2016, 2018) **FMRP** (Castren et al., 2005; Tervonen et al., 2009; Callan et al., 2010; Saffary and Xie, 2011; Jeon et al., 2014) **TRIM-32 (Brat, mei-P26,NHL-2)** (Bello, 2006; Betschinger et al., 2006; Lee et al., 2006; Bowman et al., 2008; Neumüller et al., 2008; Hammell et al., 2009; Schwamborn et al., 2009; Loewen et al., 2018) **Hu Proteins** (Marusich et al., 1994; Dobashi et al., 1998; Kasashima et al., 1999; Anderson et al., 2000; Akamatsu et al., 2002, 2005; Perrone-Bizzozero and Bolognani, 2002; Kraushar et al., 2014) **Pumilio** (Burow et al., 2015; Zahr et al., 2018) **ESCRT Factors** (Mochida et al., 2012) **DDX6** (Nicklas et al., 2015) **RBM4** (Tarn et al., 2016; Su et al., 2017) **CCR4-NOT** (Koch et al., 2014) **IPO-8** (Nganou et al., 2018) **Hsp-90** (Quintá et al., 2010)	**FMRP** (La Fata et al., 2014) **IPO-8** (Nganou et al., 2018) **RBM4** (Dhananjaya et al., 2018)	**FMRP** (Dockendorff et al., 2002; Ivanco and Greenough, 2002; Morales et al., 2002; Michel, 2004; Pan et al., 2004; Li, 2009; Doll and Broadie, 2015; Wang et al., 2015; Zimmer et al., 2017; Shen et al., 2019) **MOV10** (Skariah et al., 2017) **Pumilio** (Menon et al., 2004, 2009; Ye et al., 2004; Vessey et al., 2010; Olesnicky et al., 2012) **ESCRT Factors** (Sweeney et al., 2006; Konopacki et al., 2016) **HPat** (Pradhan et al., 2012) **hnRNP Q** (Chen et al., 2012) **IPO-8** (Nganou et al., 2018) **Hsp-90** (Quintá et al., 2010; Benitez et al., 2014)	**AGO2** (Rajgor et al., 2017, 2018; Paradis-Isler and Boehm, 2018) **GW182** (Rajgor et al., 2018) **MOV10** (Ashraf et al., 2006; Banerjee et al., 2009; Kute et al., 2019) **FMRP** (Jin et al., 2004; Michel, 2004; Pan et al., 2004; Muddashetty et al., 2011; Sudhakaran et al., 2014; Dear et al., 2017; Doll et al., 2017; Wang et al., 2018) **CNOT7** (McFleder et al., 2017) **Hpat** (Pradhan et al., 2012) **Pumilio** (Menon et al., 2004)

*Several miRISC proteins are known to play an important role during different stages of neuronal development. For some proteins (e.g., AGO2, GW182, FMRP, MOV10), this role is shown to be mediated via miRISC modulation. In others, the potential role of miRISC interaction in regulating neurodevelopment is not understood*.

Finally, it is important to highlight the role of the dynamic composition of miRISC in normal neuronal development as well as its potential implication in disorders of neuronal development. Indeed, defective translational regulation is a common feature of multiple neurodevelopmental and neuropsychiatric disorders (Kapur et al., 2017; Chen et al., 2019; Neelagandan et al., 2019; Zhou et al., 2019). The role of miRNA mediated regulation of gene expression has been widely studied in these disease conditions. Most of these studies have focused on the altered miRNA levels in neuronal disorders. While this is informative, we argue that it is also important to investigate the composition of miRISC under these disease conditions. Fragile X syndrome (FXS) could be an ideal case study, where the loss of FMRP (a well-studied miRISC associated RBP) leads to a significant defect in neuronal development (Jin et al., 2004; Bassell and Warren, 2008). Here, the role of FMRP regulating the miRISC function to the pathophysiology of FXS is tentatively established (Edbauer et al., 2010; Muddashetty et al., 2011; Kute et al., 2019; Ramakrishna and Muddashetty, 2019). FMRP and many other RBPs are likely to play a similar role in the pathophysiology of neurodevelopmental disorders due to their association with miRISC. Further studies are needed to elucidate this link which may help in the broader molecular understanding of neurodevelopmental disorders.

Recent SFARI study has identified missense mutations in miRISC proteins AGO1, TNRC6B, and CNOT3 among patients diagnosed with Autism Spectrum Disorders (Chen et al., 2019) Patients with microdeletions of chromosomal region 1p34.3 encompassing the AGO1 and AGO3 genes also show several neurodevelopmental defects (Tokita et al., 2015). Similarly, studies have also identified a crucial role of miRISC in neurodegenerative disorders. Reduced expression of AGO2 was observed in the brain of the rodent model for multiple sclerosis. This was further correlated with reduced AGO2-GW182 interaction and dysregulated miRISC assembly (Lewkowicz et al., 2015). In fact, miRISC protein GW182 was first identified from the serum auto-antibodies of patients with neuropathic symptoms (Eystathioy et al., 2002, 2003), and changes in its levels are implicated in Alzheimer's disease (Rouillard et al., 2016; Badhwar et al., 2017). Altered localization and trafficking of miRISC was recently reported in a cell culture model of amyotrophic lateral sclerosis (ALS) (Gershoni-Emek et al., 2018). Moreover, recent studies highlighting the interactions of miRISC components with proteins involved in neurodegenerative disorders, such as HTT, FUS, and Ataxin points toward an important role of miRISC composition in the pathology of these disease which should be investigated in detail (Savas et al., 2008; McCann et al., 2011; Zhang T. et al., 2018). Further, the above-mentioned studies showed that mutant versions of HTT and FUS impair miRNA mediated silencing, providing further evidence for the role of miRISC composition in driving neurodegenerative disorders (Savas et al., 2008; Zhang T. et al., 2018). All these studies indicate that it is not miRNA alone, but the composition of miRISC has a great significance in the pathophysiology of disorders of the nervous system. There is a great need and enormous potential for understanding the role of the dynamic composition and function of miRISC in health and disease-related to the development of the nervous system.

## Author Contributions

BN, SR, and RM reviewed the literature, conceptualized, and wrote the manuscript.

### Conflict of Interest

The authors declare that the research was conducted in the absence of any commercial or financial relationships that could be construed as a potential conflict of interest.
